# Genome Replication in *Thermococcus kodakarensis* Independent of Cdc6 and an Origin of Replication

**DOI:** 10.3389/fmicb.2017.02084

**Published:** 2017-10-27

**Authors:** Alexandra M. Gehring, David P. Astling, Rie Matsumi, Brett W. Burkhart, Zvi Kelman, John N. Reeve, Kenneth L. Jones, Thomas J. Santangelo

**Affiliations:** ^1^Department of Biochemistry and Molecular Biology, Colorado State University, Fort Collins, CO, United States; ^2^Department of Biochemistry and Molecular Genetics, University of Colorado School of Medicine, Aurora, CO, United States; ^3^Department of Microbiology, Ohio State University, Columbus, OH, United States; ^4^Biomolecular Labeling Laboratory, Institute for Bioscience and Biotechnology Research, National Institute of Standards and Technology and the University of Maryland, Rockville, MD, United States

**Keywords:** archaea, DNA replication, *Thermococcus kodakarensis*, recombination, DNA origins

## Abstract

The initiation of DNA replication is typically tightly regulated by proteins that form initiation complexes at specific sequences known as replication origins. In Archaea and Eukaryotes, Cdc6, a near-universally conserved protein binds and facilitates the origin-dependent assembly of the replicative apparatus. TK1901 encodes Cdc6 in *Thermococcus kodakarensis* but, as we report here, TK1901 and the presumed origin of replication can be deleted from the genome of this hyperthermophilic Archaeon without any detectable effects on growth, genetic competence or the ability to support autonomous plasmid replication. All regions of the genome were equally represented in the sequences generated by whole genome sequencing of DNA isolated from *T. kodakarensis* strains with or without TK1901, inconsistent with DNA initiation occurring at one or few origins, and instead suggestive of replication initiating at many sites distributed throughout the genome. We were unable to generate strains lacking the recombination factors, RadA or RadB, consistent with *T. kodakarensis* cells, that are oligoploid (7–19 genomes per cell), employing a recombination-based mechanism of DNA replication. Deletion of the previously presumed origin region reduced the long-term viability of cultures supporting the possibility that retaining an origin-based mechanism of DNA initiation provides a survival mechanism for stationary phase cells with only one genome.

## Introduction

DNA replication is fundamental for cellular life and although there are differences in the details, the initiation of genome replication has common features in *Bacteria, Archaea*, and *Eurkaryotes*. An initiator protein or protein complex recognizes and assembles at one (all *Bacteria* and some *Archaea*) or multiple sites (some *Archaea* and all *Eukaryotes*) that function as origins of replication (Jacob et al., 1963). Under exceptional circumstances, initiator protein-independent genome replication, termed *r*ecombination-*d*riven DNA *r*eplication initiation (RDR; also termed inducible and constitutive stable DNA replication) has been documented in *Bacteria*, but such mechanisms support—at best—only minimal cell growth (Ogawa et al., 1984; Masai et al., 1994; Kogoma, 1997; Maduike et al., 2014). It was surprising then when Hawkins et al. (2013) proposed that RDR not only facilitates genome replication but supports faster-than-wild type growth of a strain of the halophilic archaeon, *Haloferax volcanii* from which they had genetically deleted all four of the recognized origins of genome replication. Consistent with RDR initiation, the recombination factor RadA was essential for viability of the origin-less strain but could be deleted from the genome of the parental, origins-containing *H. volcanii*.

Given the established and convincingly large body of evidence that archaeal genomes have defined origins recognized and bound by initiator proteins (Matsunaga et al., 2001, 2007, 2010; Norais et al., 2007; Wigley, 2009; Kawakami and Katayama, 2010; Beattie and Bell, 2011; Scholefield et al., 2011; Pelve et al., 2013), the proposal that RDR supports rapid growth in an archaeon (Hawkins et al., 2013) is unique and challenging. Most archaea encode replication initiator proteins that are homologous to eukaryotic initiation factors Orc1 and Cdc6, and one or more Cdc6-encoding genes are present in almost all sequenced archaeal genomes, usually located adjacent to a known or predicted origin(s) of replication (Robinson and Bell, 2005; Barry and Bell, 2006; Dueber et al., 2011; Bell, 2012; Makarova and Koonin, 2013; Samson et al., 2013; Arora et al., 2014; Wu et al., 2014; Cossu et al., 2015). An increase in the number of Cdc6 proteins is often positively correlated with the number of replication origins (Samson et al., 2013); *H. volcanii* encodes fourteen Cdc6 proteins that function at three chromosomal origins and an integrated viral origin (Norais et al., 2007). Some species are reliant on a single encoded, or only a single-functional Cdc6 protein to initiate replication, and the remaining Cdc6 isoforms are predicted to play roles in transcription regulation, recombination, replication restart, or negative regulation of replication initiation (Ausiannikava and Allers, 2017).

To address the roles of Cdc6, presumptive origin sequences, and the potential of RDR to support rapid growth of archaeal strains, we took advantage of a procedure that permits the precise deletion of non-essential genome sequences and provides strong statistical evidence for essential genes in the hyperthermophilic archaeon *Thermococcus kodakarensis* (Hileman and Santangelo, 2012). Employing similar techniques, several essential and some surprisingly non-essential genes have already been identified revealing unanticipated features in archaeal DNA replication (Li et al., 2010, 2014; Pan et al., 2011, 2013; Cubonova et al., 2013). Bioinformatic analysis including GC-skew and Z-curve analysis predict only one origin of replication located directly adjacent to the gene encoding Cdc6 in *T. kodakarensis* (Fukui et al., 2005; Ojha and Swati, 2010; Cossu et al., 2015). Most members of the *Thermococcales*, including *T. kodakarensis*, encode only one identifiable Cdc6 protein (Makarova and Koonin, 2013). We now report that Cdc6 and the adjacent previously-presumed origin of replication can be deleted from *T. kodakarensis* with no detectable consequences for viability, growth, genetic competence, or plasmid maintenance. Data obtained by whole genome sequence and marker frequency analyses (Xu et al., 2012), coupled with the apparent essentiality of RadA and RadB, provide strong evidence that *T. kodakarensis* normally employs a RDR mechanism for initiation that occurs at many sites around the genome.

## Results

### Construction of *T. kodakarensis* Δ*cdc6*

The procedure employed to delete genes from the *T. kodakarensis* genome permits a statistical definition of essentiality (Hileman and Santangelo, 2012). Plasmids are constructed and used to transform a parental strain (here *T. kodakarensis* TS559) so that the target locus is flanked by two sets of direct repeats. Spontaneous recombination in this intermediate strain between one set of the repeats results in the markerless deletion of the target locus, whereas an equally-probable recombination between the second set of repeats regenerates the parental strain. When only the parental strain is recovered, after screening >30 isolates generated from at least two independently-constructed intermediate strains, the target locus is defined operationally as essential for *T. kodakarensis* viability under our laboratory conditions.

**Figure 1:**
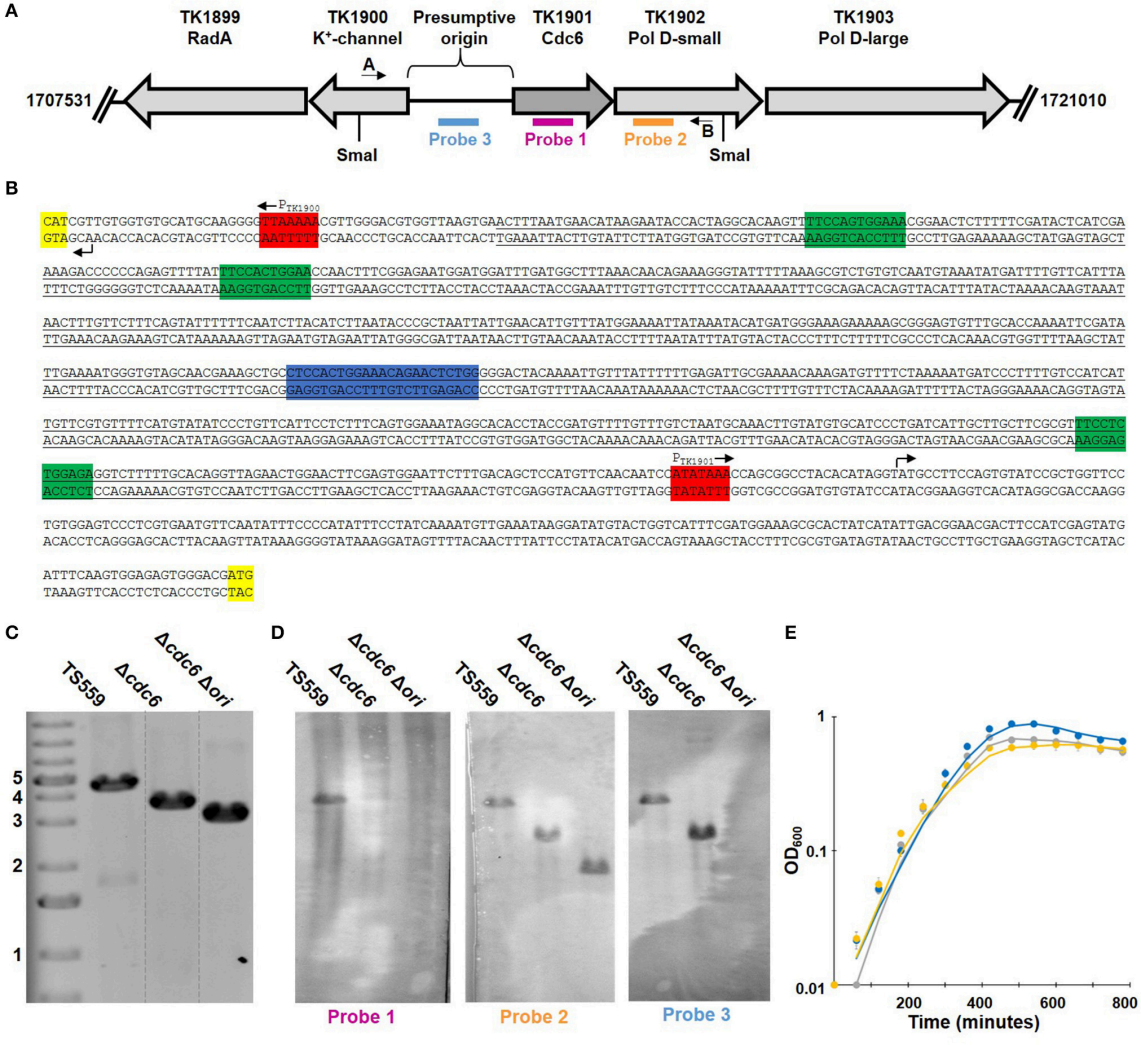
Deletion of TK1901 and the presumptive origin of replication from *T. kodakarensis* is non-phenotypic. **(A)** Organization of the *T. kodakarensis* genome surrounding TK1901. The locations of sequences used as primers in PCRs, probes in Southern blotting and SmaI recognition sites are shown. **(B)** Sequence of the presumptive origin region of *T. kodakarensis* with the ORB (blue), mini-ORBs (green), promoters (red), and the first codon of the gene (yellow) marked. The full sequence of *cdc6* plus the underlined nucleotides were deleted from the genome in *T. kodakarensis* Δ*cdc6* Δ*ori*. **(C)** PCR generate amplicons confirm deletion of TK1901, as well as TK1901 and the presumptive origin from *T. kodakarensis* Δ*cdc6* and Δ*cdc6* Δ*ori*, respectively. **(D)** Southern blots of SmaI-digested genomic DNA from *T. kodakarensis* TS559, Δ*cdc6*, and Δ*cdc6* Δ*ori* confirm deletion of *cdc6*, as well as *cdc6* and the presumptive origin, respectively. **(E)** Deletion of *cdc6* or *cdc6/ori* does not affect laboratory growth of *T. kodakarensis* TS559 (gray), Δ*cdc6* (blue), and Δ*cdc6* Δ*ori* (yellow). Error bars report standard error of the mean of three biological replicates grown in triplicate.

TK1901-TK1902-TK1903 (encoding Cdc6, DNA polymerase D small and large subunits, respectively) form an operon (Jager et al., 2014) and essentiality has been previously established for TK1902 and TK1903 (Figure 1; Cubonova et al., 2013). Surprisingly, this was not true for TK1901, the only gene in *T. kodakarensis* that encodes a recognizable Cdc6 homolog. The design of the plasmid constructed to delete TK1901 ensured retention of the upstream promotor and so continued expression of TK1902-TK1903, and avoided deletion of any sequences in the adjacent ~900 bp region predicted to contain the origin of replication based on homology with the origin region in *Pyrococcus furiosus*, a related member of the *Thermococcales* (Figure 1; Farkas et al., 2011; Cossu et al., 2015).

The presence of the TK1901 deletion was confirmed by diagnostic PCR and Southern blotting in two independent isolates (Figure 1). Amplicon sequencing confirmed that the 1,248 bp deletion extended precisely from the ATG-start codon to TGA-stop codon of TK1901, and this was subsequently re-confirmed by deep-sequencing (see below) of the entire genome of one isolate, designated *T. kodakarensis* Δ*cdc6*. This isolate was phenotypically similar to the parental *T. kodakarensis* TS559 strain: cultures grew at similar rates and reached the same final cell densities (Figure 1) and *T. kodakarensis* Δ*cdc6* was genetically competent and supported the autonomous replication of pTN1-based plasmids (Santangelo et al., 2008).

### Marker frequency analysis of genomic DNA

**Figure 2:**
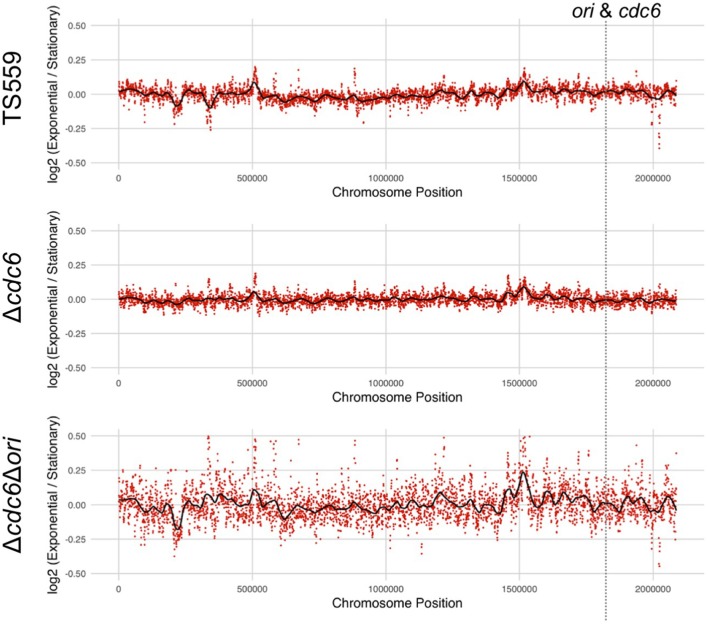
Marker frequency analysis of DNA sequence reads from *T. kodakarensis* strains fails to identify a defined origin(s) of replication. The log_2_ ratio of each nucleotide in sequences from exponentially growing cells divided by sequence from stationary phase cells is shown in each panel. Frequencies were calculated using 1 Kbp intervals (red dots) with a sliding window of 500 bp generating the average frequency shown in black. The location of the *ori*-*cdc6* region is indicated.

Regions adjacent to an origin(s) are over-represented in growing cells, and marker frequency analyses comparing the number of sequencing reads across the genome has been used to identify replication origin(s) in many archaeal genomes (Andersson et al., 2010; Hawkins et al., 2013; Pelve et al., 2013). Genomic DNA was therefore isolated, fragmented, and deep-sequenced from growing and stationary phase cultures of *T. kodakarensis* TS559 and Δ*cdc6*. The sequences obtained confirmed the deletion of TK1901 but, in repeated experiments, all regions of the genome were equally represented in the DNA reads from both growing and stationary phase cells of both *T. kodakarensis* TS559 and Δ*cdc6* (Figure 2 and Supplementary Figure 1). Given this unanticipated result, to provide confidence in the laboratory and computational procedures, the experiments were repeated with genomic DNAs from *Escherichia coli* MG1655 and *P. furiosus* strain JFW02(Farkas et al., 2012), species with established origins of replication. Quantification of the whole genome sequencing (WGS) reads clearly and correctly identified the origin loci established in the genomes of *E. coli* and *P. furiosus* (Supplementary Figure 2; Burland et al., 1993; Farkas et al., 2012).

### Construction of *T. kodakarensis* Δ*cdc6* Δ*ori*

Using the same markerless-genome modification techniques, the presumed origin sequences (Ojha and Swati, 2010) were easily deleted in *T. kodakarensis* Δ*cdc6* (Figure 1). All the bioinformatically identified mini-origin recognition boxes (mini-ORBs; green) and one full ORB (blue) were deleted while retaining the promoters (red), transcription start sites (arrows), and translation start sites (yellow) for TK1902-1903, and for TK1900 (Figure 1). Both amplicon- and whole genome deep-sequencing confirmed the precision of the deletion. A representative isolate, designated *T. kodakarensis* Δ*cdc6* Δ*ori*, was phenotypically indistinguishable from *T. kodakarensis* TS559 and Δ*cdc6*. All three strains grew at the same rate, achieved the same final cell densities, were genetically competent and supported plasmid replication. Quantification of WGS reads also failed to identify any preferred origin(s) sequences and indicated that replication was initiated at many sites around the genome of *T. kodakarensis* Δ*cdc6* Δ*ori* (Figure 2).

### Spontaneous genome deletion and inversion

**Figure 3:**
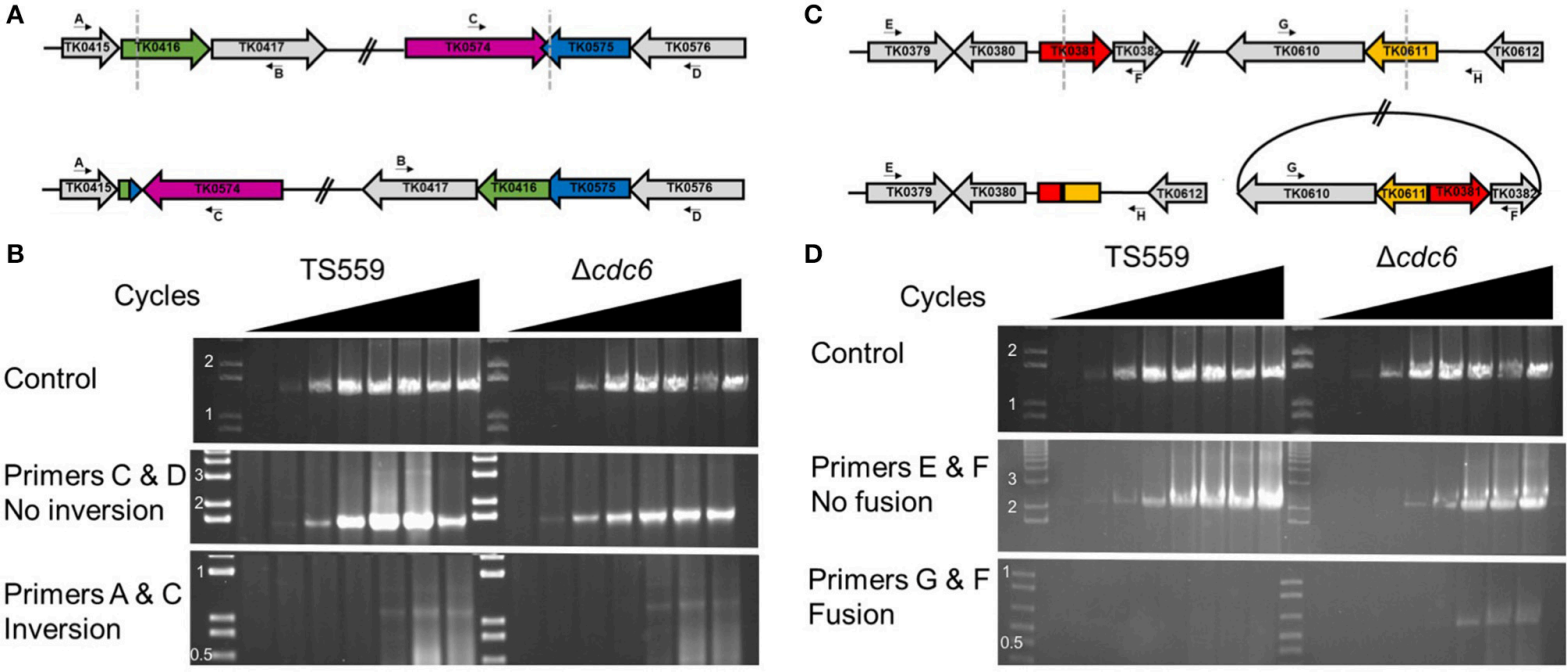
Semi-quantitative PCR supports large-scale genomic rearrangements. **(A)** Representations of TS559 (top) and inverted (bottom) genome structures. The end points of the inversion events are marked with dotted gray lines in the TS559 genome representation. **(B)** Semi-quantitative PCRs demonstrate the inversion genome rearrangements identified from the WGS data. The presence of the inversion was identified in <10% of the samples. **(C)** Representations of TS559 (top) and fusion-event (bottom) genome structures. The end points of the fusion events are marked with dotted gray lines in the TS559 genome representation. **(D)** Semi-quantitative PCRs demonstrate the fusion event identified from the WGS data in <10% of the samples.

Although, the WGS reads did not identify origins of replication, with >2,000x genome coverage, they did identify spontaneous recombination events at two locations in subpopulations (<10%) of *T. kodakarensis* TS559 cells (Figure 3). The recombinations inverted an ~150 kbp region or excised ~100 kbp, and these events resulted in small spikes and dips in the marker frequency analyses at sites previously established to contain vestigial prophage TKV2 and TKV3 genomic sequences (Figure 3; Fukui et al., 2005; Tagashira et al., 2013). Only a small number of sequences were obtained that extended across the sites of recombination, (Figure 3) but these were more prevalent in DNAs isolated from growing than from stationary phase cells. Based on PCR amplicons, these recombination events also occurred in *T. kodakarensis* Δ*cdc6* and Δ*cdc6* Δ*ori*. As deletion of TKV3 severely hinders growth (Tagashira et al., 2013) and several presumably essential genes are within the deleted sequences, these recombination events are likely lethal and prevent the effected cells from contributing to continued culture growth.

### Mutations acquired by *T. kodakarensis* since isolation

*T. kodakarensis* KOD1 has been the focus of continuous research since its isolation in 1994 (Morikawa et al., 1994). It seemed possible therefore that the cdc6- and origin-independent replication of *T. kodakarensis* TS559 might result from mutations acquired and selected during laboratory culture. The genome sequence determined here for *T. kodakarensis* TS559 was therefore compared with that published for *T. kodakarensis* KOD1 (Fukui et al., 2005). All the changes known to have been intentionally introduced to generate *T. kodakarensis* TS559 from *T. kodakarensis* KOD1 were present, and although there were no large genome rearrangements, an additional 35 single nucleotide differences were identified. Some of these changes are within open reading frames, but none would be predicted to radically change DNA replication or recombination (Table 1).

**Table 1:** Differences in the genome sequences of the *T. kodakarensis* KOD1 and TS559.

**Position^a^**	**TK Gene^b^**	**Operon**	**DNA change**	**Protein change**	**Annotated/Putative function^b^ (result of the mutation)**
37106	0042	0038 → 0050	A → G	K185R	Flagellin
76583	0090	0086 → 0090	A → C	Q187H	Putative S-layer function
96733	0119	0119 → 0122	A → G	E183G	α-subunit proline dehydrogenase
201247	0238	0237 → 0241	A → G	E215G	Nitrilase; C-H bond hydrolase
229773	0275	0279 → 0274	C → T	R351K	argD; acetyl-lysine amino transferase
327973	0392	0384 → 0393	A → G	N.C.^c^	Hypothetical
327976	0392	0384 → 0393	C → A	N.C.	Hypothetical
343671	0415	0410 → 0419	G → A	G24E	Hypothetical
538367	0634	0631 → 0638	G → A	N.C.	Chemotaxis methyl-acceptor
785924	Inter^d^		C → G	–	
785946	0901	0902 → 0901	ΔΔG	S115fs^e^	F-subunit RNA polymerase (frameshift extends the ORF –S^*^ to –IDEYRPLE^*^ at C-terminus)
898031	1021	1020 → 1021	ΔΔA	T800fs	Hef nuclease (frameshift extends the ORF –TGTLR^*^ to –QAPYVEEEDKA^*^ at C-terminus)
912171	1039	1039 → 1038	T → C	K342R	Cyclic 2′3′-diphosphoglycerate synthetase
914113	1041	1041 → 1042	C → G	D132E	Transcription regulator with H-T-H domain
1084046	1236	1236	C → G	A209P	AAA+ family ATPase
1124276	1285	1285	C → T	G102D	Transcription regulator; LysR/AsnC with HTH domain
1124363	1285	1285	A → C	L73R	Transcription regulator; LysR/AsnC with HTH domain
1127248	inter	tRNA	↑^f^T	–	
1160792	1315	1315	A → C	F581V	Phosphoadenosine phosphosulfate reductase
1160804	1315	1315	A → C	F577V	Phosphoadenosine phosphosulfate reductase
1252468	1428	1429 → 1429	A → G	V20A	Metal-dependent RNase with KH-domain
1361362	1554	1554	↑C	P412fs	Cellulose synthetase; glycosyl transferase (frameshift changes CTSWFSSLRGLCTP^*^ to -LYFMVFVLAGVVYTMRGLTKLLIGKLTWEKT QFRT^*^ at C-terminus)
1524161	1729	1729 → 1730	↑A	L211fs	Mannosyl transferase (frameshift results in in-frame ORF fusion with TK1730; TK1729 –NGEPATLC^*^ to TK1729 –LKWGARYIV-TK1730)
1580984	1774	1770 → 1776	ΔΔA	T1069fs	Amylopullanase (frameshift extends the ORF–NHHDYYNHIPRRRRKWQRIHHYQHLPRHRRW^*^ to TTTTTTTTSPGGGGSGSGTTTSTSPGT GGGEEGGGICGPAFLVGLAVVPLLLRRRR^*^ at C-terminus; does not overlap TK1775)
1585144	inter		ΔΔG	–	
1596662	1789	1789 → 1787	T → C	E108G	KaiC domain; recA-like ATPase
1743876	1932	1932 → 1930	↑T	N30fs	KaiC domain; ABC-family ATPase (frameshift results in 31 in-frame amino acids then ^*^)
1824228	2030	2030	T → C	F70L	ACT-domain; amino acid metabolism regulator
1824230	2030	2030	T → A	F70L	ACT-domain; amino acid metabolism regulator
1824411	2030	2030	ΔΔG	R131fs	ACT-domain; amino acid metabolism regulator (frameshift changes GRNKQDLHSHRWNALNR DIWQNKDNQRLQEAHTPHT^*^ to EETSKIYIVIDGTLSTETFGKIKTIRGFKRLILHTPEKDKEKFVCNYCEVKYCPKRVLLESLTTQR^*^ at C-terminus
1828936	inter		↑G	–	
1860427	2069	2072 → 2066	↑C	V16fs	α-subunit of cytosolic NiFe hydrogenase (frameshift changes –GRGQGRR to -VEGKGGV^*^ at aa 138)
2011218	2222	2222	T → G	T242P	ATPase
2050612	2262	2261 → 2263	G → T	R119L	PIN domain, likely VAPC toxin
2078803	2298	2298 → 2299	C → G	P247A	Anaerobic ribonucleoside reductase class III

a *Genome position 0 was defined in (27)*.

b *Numerical gene designations, TKxxxx, and annotated functions based on (27)*.

c *No change*.

d *Intergenic region*.

e *Frame shift*.

f *Nucleotide insertion*.

### Why are Cdc6 and the origin-sequences retained?

**Figure 4:**
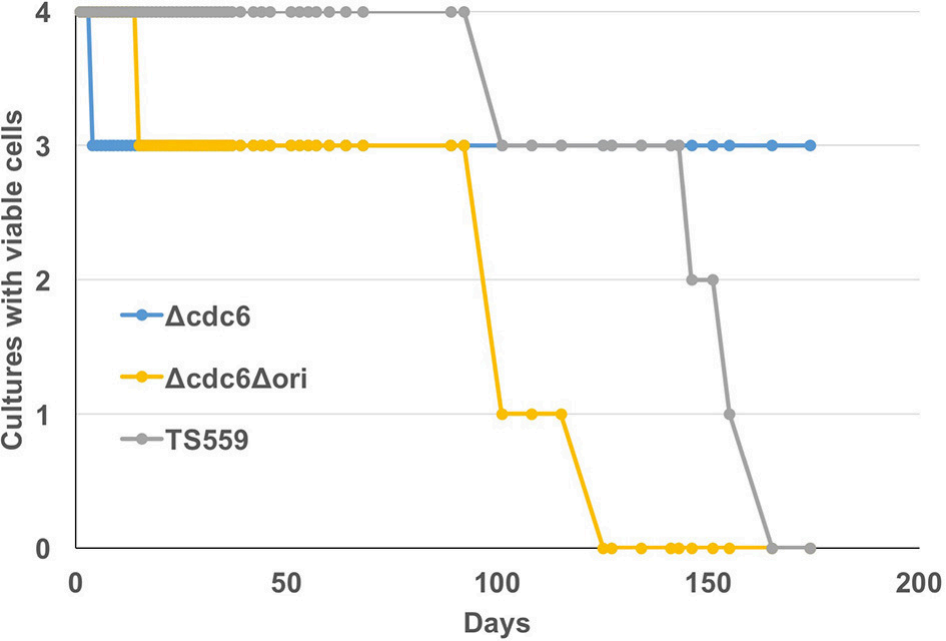
Presence of the origin region increases long-term viability. Four biological replicates of *T. kodakarensis* TS559 (gray), Δ*cdc6* (blue), and Δ*cdc6*Δ*ori* (yellow) were grown to stationary phase and incubation was continued without additions to the medium for >170 days. Aliquots were removed at intervals and used to inoculate fresh growth media. The number of cultures with viable cells that generated progeny cultures is plotted against days of incubation at 85°C.

*T. kodakarensis* is oligoploid (7–19 genomes/cell; Spaans et al., 2015) and this is consistent with the use of RDR. Nutrient-stress, the absence of defined DNA segregation strategies, and the potential for continued cell division without DNA replication may occasionally result in *T. kodakarensis* cells with only one genome. Such cells would be unable to restart growth by RDR initiation but could do so if a cdc6-oriC system of replication initiation was also available. To evaluate whether retention of *cdc6* or presumptive origin sequences promotes long-term viability, aliquots were taken from stationary phase cultures maintained at 85°C for extended periods without nutrient addition, and assayed for viability. Extended (several months) incubation at elevated temperatures in nutrient poor conditions was predicted to deplete energy reserves, introduce stress into the genome, and potentially reduce ploidy as genomes were consumed to provide nutrients. Cells in cultures of *T. kodakarensis* TS559 and Δ*cdc6*, strains with the presumed origin (oriC) region, were viable for ~40 days longer than cells in cultures of *T. kodakarensis* Δ*ori* (Figure 4). Retention of Cdc6, however, did not influence long-term viability.

### TK1899 (RadA) and TK2231 (RadB) are essential genes

RadA was required for growth—presumably by RDR initiation—of the origin-deleted halophilic *H. volcanii* strains but non-essential in the parental, origins-containing strain (Hawkins et al., 2013). Despite repeated attempts with different transforming DNA strategies, we were unable to generate *T. kodakarensis* strains with RadA (TK1899) or RadB (TK2231) deleted, regardless of the retention or absence of *cdc6* and/or origin sequences.

## Discussion

TK1901 encodes what appears to be a fully functional Cdc6 protein, with intact Walker A and Walker B motifs, DNA, and ATP-binding domains. TK1901 is co-transcribed with TK1902 and TK1903, essential genes that encode the subunits of DNA polymerase D (Jager et al., 2014) and is located immediately adjacent to a region with sequences very similar to those of the origin of replication in *P. furiosus*. Nevertheless, the results reported establish conclusively that TK1901 and so Cdc6 are not required for *T. kodakarensis* viability and the absence of Cdc6 has no detectable effect on laboratory growth, genetic competence, or the ability to support autonomous plasmid replication. Equally surprising, the previously presumed origin of replication can also be deleted without any detectable phenotypic consequence. Consistent with Cdc6 and oriC having no essential roles, marker frequency analyses of WGS data obtained from cultures of *T. kodakarensis* TS559, Δ*cdc6*, and Δ*cdc6* Δ*ori* provided no evidence for origin-dependent replication initiation, even when the origin and the recognition protein were both present. Given the depth of the WGS, any regional over-representation of reads, consistent with initiation at as many as 5 separate locations would have been detected, but this was not the case. In contrast, the WGS results argue for genome replication in *T. kodakarensis* TS559 being initiated at many sites distributed around the genome, consistent with the proposal for RDR-dependent genome replication in the *H. volcanii* strain with all origins of replication deleted (Hawkins et al., 2013). We extend this proposal to suggest that RDR supports growth of *T. kodakarensis* despite the presence of a predicted origin. In support of this assertion, despite a considerable effort, we were unable to generate *T. kodakarensis* strains with TK1899 (RadA) or TK2231 (RadB) deleted. In *E. coli*, long-lived R-loops accumulate in strains lacking RNase H, and these R-loops can facilitate initiator protein (DnaA) independent constitutive stable DNA replication, but their growth is very slow (Masai et al., 1994; Masai and Arai, 1996; Maduike et al., 2014). The *T. kodakarensis* strains investigated here all express TK0805, the gene that encodes RNase HII (Heider et al., 2017) and thus R-loop accumulation is unlikely to be responsible for origin-independent genome replication in *T. kodakarensis*.

Employing RDR for genome replication could also explain why *T. kodakarensis* is atypically naturally competent and so amenable to genetic manipulation. Additional features of *T. kodakarensis* are consistent with RDR. In genomes replicated from distinct origins, highly expressed genes are transcribed predominantly in the same direction as replication fork movement (Smith et al., 2007; Andersson et al., 2010; Paul et al., 2013; Cossu et al., 2015). But, if bidirectional replication was initiated from the previously presumed origin in *T. kodakarensis*, 628 transcripts would be transcribed with, and 626 would be transcribed against the direction of the replisome movement (Fukui et al., 2005; Jager et al., 2014; Cossu et al., 2015). A computational search also failed to identify any location, and so a putative origin, anywhere on the genome that would result in transcription and DNA replication occurring predominantly in the same direction (Cossu et al., 2015). Often, when cloned, an origin of genome replication will still function and can be used to construct self-replicating plasmids. This is the case for the origin of replication cloned from *P. furiosus* (Farkas et al., 2011) and the generated plasmids replicate not only in the cytoplasm of *P. furiosus* but also in *T. kodakarensis*. In contrast, cloning many variants of the very similar presumed origin region from the *T. kodakarensis* genome did not result in a replicating plasmid.

RDR initiation requires the retention of more than one genome, and it is now well-established that members of the *Euryarchaeota* including *T. kodakarensis*, are naturally oligoploid (Breuert et al., 2006; Hildenbrand et al., 2011; Spaans et al., 2015). To date, however, there is no evidence for precise genome segregation strategies suggesting that growing cultures will naturally produce cells with varying ploidy—including monoploid cells. If cells occur with only one genome, then the presence of an origin-dependent initiation module would provide a survival mechanism. With this in mind, we demonstrated that cultures of *T. kodakarensis* TS559 and Δ*cdc6* did retain viability longer than *T. kodakarensis* Δ*cdc6* Δ*ori*. The signature of an origin suggests that *T. kodakarensis* has relied on an origin-dependent replication strategy during its evolutionary history (Ojha and Swati, 2010). Retention of Cdc6 did not influence survival under identical conditions, suggesting that Cdc6 may not be necessary for use of the presumptive origin sequences.

The presence of ≥~20 genomes per *T. kodakarensis* cell (Spaans et al., 2015) raises challenging questions as to how they are all accommodated and replicated within a generation time of ~40 min. As established for the DNA clamp (PCNA1; >1,000 molecules/cell; Kuba et al., 2012; Pan et al., 2013), at minimum, the replisome components must be present at very high levels and maybe this also facilitates simultaneous replication from many sites around the *T. kodakarensis* genome. How the replicative apparatus is assembled and how simultaneous rounds of replication are prohibited or accommodated during rapid growth remain outstanding questions.

## Materials and methods

### Growth of microorganisms

*T. kodakarensis* strains were grown in artificial seawater (ASW) supplemented with 5 g/L of both *y*east extract and *t*ryptone (YT) and 2 g/L of sulfur (S°) or 5 g/L sodium pyruvate (Pyr) at 85°C. *P. furiosus* strain JFW02 (Farkas et al., 2012) was grown at 95°C as described using maltose as a carbon source (Adams et al., 2001). *E. coli* strain MG1655 was grown in Luria-Bertani (LB) broth at 37°C. The growth of cultures was measured by increases in optical density at 600 nm (OD_600_). *T. kodakarensis* cultures were harvested at an OD_600_ of 0.2 (early exponential) and 0.6 (late-exponential) while the stationary phase cells were harvested at an OD_600_ of ~1.0. In Figure 4, four independent cultures of each *T. kodakarensis* strain were maintained at 85°C in sealed vessels with no additions to the cultures over ~180 days. Loss of culture viability was defined as the inability of aliquots, sampled multiple times over 3 days, to initiate culture growth when inoculated into fresh medium. The inability of 9 or more individual aliquots, removed from cultures over 3 consecutive days, to support outgrowth confirmed that these cultures had lost all viable CFUs.

### Strain construction of *T. kodakarensis*

Standard procedures (Hileman and Santangelo, 2012) were used to construct plasmids, pOSUTK1901B and pJG4 respectively, that were used to delete TK1901 or TK1901 plus the origin sequences from *T. kodakarensis* TS559. In the resulting strains, *T. kodakarensis* Δ*cdc6* and Δ*cdc6* Δ*ori*, the Cdc6 encoding sequence [TK1901; 1,248 bp] was deleted but the promoter for the TK1901-TK1903 operon was retained to sure expression of TK1902-1903. Similarly, in *T. kodakarensis* Δ*cdc6* Δ*ori*, the putative origin (640 bp) with one origin recognition box (ORB) and three mini-ORBs were deleted, but the promoters for TK1901-1903 and TK1900 were retained (Figure 1). Use of the same procedures, and plasmids designed to precisely delete the TK1899 (RadA) and TK2231 (RadB) sequences, did not generate viable strains with the desired deletions.

### Isolation of genomic DNA

Genomic DNA was isolated from all strains as described (Santangelo et al., 2007). Cells pelleted from cultures at the designated OD_600_ were resuspended in 10% (w/v) sucrose, 20 mM Tris-HCl pH 8.0, and 5 mM EDTA. SDS (2% final concentration) and proteinase K (0.25 mg/ml) were added to the resulting lysate and the mixture was incubated for 1 h at 55°C. NaCl (1 M final concentration) was then added, the mixture was chilled, centrifuged, and an equal volume of isopropanol was added to the clarified supernatant. The precipitated nucleic acids were pelleted, resuspended with 10 mM Tris-HCl pH 8.0, 50 μg of RNase A added and incubation continued at 37°C for 30 min. The DNA remaining was further purified by repeated phenol/chloroform/isoamyl alcohol (25:24:1) extraction and an alcohol precipitation.

### Southern blotting

The procedure used has been previously described (Cubonova et al., 2013). In Figure 1, TK1901 (Probe 1, pink) is only detectable in strain TS559 whereas origin sequences (Probe 3, blue) are detectable in strains TS559 and Δ*cdc6*, but not strain Δ*cdc6* Δ*ori*; probe 3 highlights a smaller product in Δ*cdc6* that reflects deletion of TK1901. Probe 2 (orange) detected the sequences encoding TK1902 in all samples and the fragment lengths identified are appropriate for the corresponding strains.

### Whole genome sequencing (WGS)

Sequencing libraries were prepared using TruSeq DNA library preparation kits (Illumina, San Diego, CA) and were subjected to WGS (pair-ended, 2 × 150 bp per read; 1 × 125 bp reads for *E. coli*) on an Illumina Hi-Seq 2000 platform (University of Colorado Denver Genomics and Microarray Core Facility). Individual genome coverages ranged from 3,300× to 7,800×.

### Comparison of genome sequences

The reference genome for *Thermococcus kodakarensis* KOD1 (https://www.ncbi.nlm.nih.gov/nuccore/NC_006624.1) was downloaded from Genbank and manually edited to account for the laboratory manipulations made in the lineage leading to *T. kodakarensis* TS559. The reference genomes for *P. furiosus* COM1 and *Escherichia coli* MG1655 were downloaded from Genbank here (https://www.ncbi.nlm.nih.gov/nuccore/CP003685 and https://www.ncbi.nlm.nih.gov/nuccore/NC_000913.3, respectively).

The *T. kodakarensis* TS559 and KOD1 genomes were aligned and differences identified by using Universal Genotyper (GATK version v2.1-8; Van der Auwera et al., 2013) and MUMmer (version 3.1; Kurtz et al., 2004). Low quality sequences, regions with <5x coverage and a small number of variants identified in DNA from only growing or stationary phase cells of the same isolate were not included. The coordinates of the RefSeq GFF file were updated to account for identified insertions and deletions, and the resulting variants annotated using snpEff (version 4.0e; Cingolani et al., 2012).

### Alignment and marker frequency analysis of whole genome sequences

An index was built for each reference genome with bowtie2-build (version 2.2.9; Langmead and Salzberg, 2012) using default settings. Illumina adaptor sequences and low quality bases (quality score <10) were trimmed from the 3′-end of each read using cutadapt (version 1.11; Martin, 2011) with reads discarded if more than half the bases were trimmed. The filtered reads were aligned to the reference genome using bowtie2 (version 2.2.9; Langmead and Salzberg, 2012) selecting the best alignment for each read. The alignment statistics are reported in Supplementary Table 1. Reads that did not align as proper pairs were treated as single end reads. For copy number estimation, each reference genome was binned into 1 kb windows with a 500 bp sliding overlap between windows using bedtools (version 2.17.0; Quinlan and Hall, 2010). For each sample, the coverage for each 1 kb was calculated as the number of sequenced bases that overlap with that window. The %G+C content of each window was calculated, and corrections made for potential bias in library preparation and sequencing due to GC content. The average coverage for each GC bin was plotted against the GC content and smoothed by a Lowess regression model. A correction factor, calculated by dividing the global mean coverage by the fitted model, was applied to each sample. Source code available at http://github.com/dpastling/plethora.

## Author contributions

JR, ZK, and TS conceived and directed the project. AG, DA, JR, and TS wrote the manuscript and prepared figures. AG, RM, BB constructed and phenotypically characterized strains. AG, DA, and KJ analyzed the WGS data.

### Conflict of interest statement

The authors declare that the research was conducted in the absence of any commercial or financial relationships that could be construed as a potential conflict of interest. Certain commercial equipment, instruments, or materials are identified in this paper to foster understanding. Such identification does not imply recommendation or endorsement by the National Institute of Standards and Technology, nor does it imply that the materials or equipment identified is necessarily the best available for the purpose.

## References

[B1] Adams M. W. Holden J. F. Menon A. L. Schut G. J. Grunden A. M. Hou C.. (2001). Key role for sulfur in peptide metabolism and in regulation of three hydrogenases in the hyperthermophilic archaeon *Pyrococcus furiosus*. J. Bacteriol. 183, 716–724. 10.1128/JB.183.2.716-724.2001 11133967 PMC94929

[B2] Andersson A. F. Pelve E. A. Lindeberg S. Lundgren M. Nilsson P. Bernander R. (2010). Replication-biased genome organisation in the crenarchaeon Sulfolobus. BMC Genomics 11:454. 10.1186/1471-2164-11-454 20667100 PMC3091651

[B3] Arora J. Goswami K. Saha S. (2014). Characterization of the replication initiator Orc1/Cdc6 from the Archaeon Picrophilus torridus. J. Bacteriol. 196, 276–286. 10.1128/jb.01020-13 24187082 PMC3911243

[B4] Ausiannikava D. Allers T. (2017). Diversity of DNA replication in the Archaea. Genes 8:56. 10.3390/genes8020056 28146124 PMC5333045

[B5] Barry E. R. Bell S. D. (2006). DNA Replication in the Archaea. Microbiol. Mol. Biol. Rev. 70, 876–887. 10.1128/MMBR.00029-06 17158702 PMC1698513

[B6] Beattie T. R. Bell S. D. (2011). Molecular machines in archaeal DNA replication. Curr. Opin. Chem. Biol. 15, 614–619. 10.1016/j.cbpa.2011.07.017 21852183

[B7] Bell S. D. (2012). Archaeal orc1/cdc6 proteins. Subcell. Biochem. 62, 59–69. 10.1007/978-94-007-4572-8_4 22918580

[B8] Breuert S. Allers T. Spohn G. Soppa J. (2006). Regulated polyploidy in halophilic archaea. PLoS ONE 1:e92. 10.1371/journal.pone.0000092 17183724 PMC1762399

[B9] Burland V. Plunkett G. Daniels D. L. Blattner F. R. (1993). DNA sequence and analysis of 136 kilobases of the *Escherichia coli* genome: organizational symmetry around the origin of replication. Genomics 16, 551–561. 10.1006/geno.1993.1230 7686882

[B10] Cingolani P. Platts A. Wang L. L. Coon M. Nguyen T. Wang le L.. (2012). A program for annotating and predicting the effects of single nucleotide polymorphisms, SnpEff. Fly 6, 80–92. 10.4161/fly.19695 22728672 PMC3679285

[B11] Cossu M. Da Cunha V. Toffano-Nioche C. Forterre P. Oberto J. (2015). Comparative genomics reveals conserved positioning of essential genomic clusters in highly rearranged *Thermococcales chromosomes*. Biochimie 118, 313–321. 10.1016/j.biochi.2015.07.008 26166067 PMC4640148

[B12] Cubonova L. Richardson T. Burkhart B. W. Kelman Z. Connolly B. A. Reeve J. N.. (2013). Archaeal DNA polymerase D but not DNA polymerase B is required for genome replication in *Thermococcus kodakarensis*. J. Bacteriol. 195, 2322–2328. 10.1128/JB.02037-12 23504010 PMC3650531

[B13] Dueber E. C. Costa A. Corn J. E. Bell S. D. Berger J. M. (2011). Molecular determinants of origin discrimination by Orc1 initiators in archaea. Nucleic Acids Res. 39, 3621–3631. 10.1093/nar/gkq1308 21227921 PMC3089459

[B14] Farkas J. Chung D. DeBarry M. Adams M. W. Westpheling J. (2011). Defining components of the chromosomal origin of replication of the hyperthermophilic archaeon *Pyrococcus furiosus* needed for construction of a stable replicating shuttle vector. Appl. Environ. Microbiol. 77, 6343–6349. 10.1128/AEM.05057-11 21784908 PMC3187180

[B15] Farkas J. Stirrett K. Lipscomb G. L. Nixon W. Scott R. A. Adams M. W.. (2012). Recombinogenic properties of *Pyrococcus furiosus* strain COM1 enable rapid selection of targeted mutants. Appl. Environ. Microbiol. 78, 4669–4676. 10.1128/AEM.00936-12 22544252 PMC3370475

[B16] Fukui T. Atomi H. Kanai T. Matsumi R. Fujiwara S. Imanaka T. (2005). Complete genome sequence of the hyperthermophilic archaeon *Thermococcus kodakaraensis* KOD1 and comparison with Pyrococcus genomes. Genome Res. 15, 352–363. 10.1101/gr.3003105 15710748 PMC551561

[B17] Hawkins M. Malla S. Blythe M. J. Nieduszynski C. A. Allers T. (2013). Accelerated growth in the absence of DNA replication origins. Nature 503, 544–547. 10.1038/nature12650 24185008 PMC3843117

[B18] Heider M. R. Burkhart B. W. Santangelo T. J. Gardner A. F. (2017). Defining the RNaseH2 enzyme-initiated ribonucleotide excision repair pathway in Archaea. J. Biol. Chem. 292, 8835–8845. 10.1074/jbc.M117.783472 28373277 PMC5448109

[B19] Hildenbrand C. Stock T. Lange C. Rother M. Soppa J. (2011). Genome copy numbers and gene conversion in methanogenic archaea. J. Bacteriol. 193, 734–743. 10.1128/JB.01016-10 21097629 PMC3021236

[B20] Hileman T. H. Santangelo T. J. (2012). Genetics techniques for *Thermococcus kodakarensis*. Front. Microbiol. 3:195. 10.3389/fmicb.2012.00195 22701112 PMC3370424

[B21] Jacob F. Brenner S. Cuzin F. (1963). On the Regulation of DNA replication in bacteria. Cold Spring Harb. Symp. Quant. Biol. 28, 329–348. 10.1101/SQB.1963.028.01.048

[B22] Jager D. Forstner K. U. Sharma C. M. Santangelo T. J. Reeve J. N. (2014). Primary transcriptome map of the hyperthermophilic archaeon *Thermococcus kodakarensis*. BMC Genomics 15:684. 10.1186/1471-2164-15-684 25127548 PMC4247193

[B23] Kawakami H. Katayama T. (2010). DnaA, ORC, and Cdc6: similarity beyond the domains of life and diversity. Biochem. Cell Biol. 88, 49–62. 10.1139/o09-154 20130679

[B24] Kogoma T. (1997). Stable DNA replication: interplay between DNA replication, homologous recombination, and transcription. Microbiol. Mol. Biol. Rev. 61, 212–238. 9184011 10.1128/mmbr.61.2.212-238.1997 PMC232608

[B25] Kuba Y. Ishino S. Yamagami T. Tokuhara M. Kanai T. Fujikane R.. (2012). Comparative analyses of the two proliferating cell nuclear antigens from the hyperthermophilic archaeon, *Thermococcus kodakarensis*. Genes Cells 17, 923–937. 10.1111/gtc.12007 23078585

[B26] Kurtz S. Phillippy A. Delcher A. L. Smoot M. Shumway M. Antonescu C.. (2004). Versatile and open software for comparing large genomes. Genome Biol. 5:R12. 10.1186/gb-2004-5-2-r12 14759262 PMC395750

[B27] Langmead B. Salzberg S. L. (2012). Fast gapped-read alignment with Bowtie 2. Nat. Methods 9, 357–359. 22388286 10.1038/nmeth.1923 PMC3322381

[B28] Li Z. Huang R. Y. Yopp D. C. Hileman T. H. Santangelo T. J. Hurwitz J.. (2014). A novel mechanism for regulating the activity of proliferating cell nuclear antigen by a small protein. Nucleic Acids Res. 42, 5776–5789. 10.1093/nar/gku239 24728986 PMC4027161

[B29] Li Z. Santangelo T. J. Cubonova L. Reeve J. N. Kelman Z. (2010). Affinity purification of an archaeal DNA replication protein network. MBio 1:e00221-10 10.1128/mBio.00221-10 20978540 PMC2962436

[B30] Maduike N. Z. Tehranchi A. K. Wang J. D. Kreuzer K. N. (2014). Replication of the *Escherichia coli* chromosome in RNase HI-deficient cells: multiple initiation regions and fork dynamics. Mol. Microbiol. 91, 39–56. 10.1111/mmi.12440 24164596 PMC3926323

[B31] Makarova K. S. Koonin E. V. (2013). Archaeology of Eukaryotic DNA replication. Cold Spring Harb. Perspect. Biol. 5:a012963. 10.1101/cshperspect.a012963 23881942 PMC3809583

[B32] Martin M. (2011). Cutadapt removes adapter sequences from high-throughput sequencing reads. EMBnet J. 17, 10–12. 10.14806/ej.17.1.200

[B33] Masai H. Arai K. (1996). Mechanisms of primer RNA synthesis and D-loop/R-loop-dependent DNA replication in *Escherichia coli*. Biochimie 78, 1109–1117. 10.1016/S0300-9084(97)86737-5 9150892

[B34] Masai H. Asai T. Kubota Y. Arai K. Kogoma T. (1994). *Escherichia coli* PriA protein is essential for inducible and constitutive stable DNA replication. EMBO J. 13, 5338–5345. 7525276 10.1002/j.1460-2075.1994.tb06868.x PMC395490

[B35] Matsunaga F. Forterre P. Ishino Y. Myllykallio H. (2001). *In vivo* interactions of archaeal Cdc6/Orc1 and minichromosome maintenance proteins with the replication origin. Proc. Natl. Acad. Sci. U.S.A. 98, 11152–11157. 10.1073/pnas.191387498 11562464 PMC58699

[B36] Matsunaga F. Glatigny A. Mucchielli-Giorgi M.-H. Agier N. Delacroix H. Marisa L.. (2007). Genomewide and biochemical analyses of DNA-binding activity of Cdc6/Orc1 and Mcm proteins in *Pyrococcus* sp. Nucleic Acids Res. 35, 3214–3222. 10.1093/nar/gkm212 17452353 PMC1904270

[B37] Matsunaga F. Takemura K. Akita M. Adachi A. Yamagami T. Ishino Y. (2010). Localized melting of duplex DNA by Cdc6/Orc1 at the DNA replication origin in the hyperthermophilic archaeon *Pyrococcus furiosus*. Extremophiles 14, 21–31. 10.1007/s00792-009-0284-9 19787415

[B38] Morikawa M. Izawa Y. Rashid N. Hoaki T. Imanaka T. (1994). Purification and characterization of a thermostable thiol protease from a newly isolated hyperthermophilic *Pyrococcus* sp. Appl. Environ. Microbiol. 60, 4559–4566. 7811092 10.1128/aem.60.12.4559-4566.1994 PMC202019

[B39] Norais C. Hawkins M. Hartman A. L. Eisen J. A. Myllykallio H. Allers T. (2007). Genetic and physical mapping of DNA replication origins in *Haloferax volcanii*. PLoS Genet. 3:e77. 10.1371/journal.pgen.0030077 17511521 PMC1868953

[B40] Ogawa T. Pickett G. G. Kogoma T. Kornberg A. (1984). RNase H confers specificity in the dnaA-dependent initiation of replication at the unique origin of the *Escherichia coli* chromosome *in vivo* and *in vitro*. Proc. Natl. Acad. Sci. U.S.A. 81, 1040–1044 6322184 10.1073/pnas.81.4.1040 PMC344759

[B41] Ojha K. K. Swati D. (2010). Mapping of origin of replication in *Themococcales*. Bioinformation 5, 213–218 21364800 10.6026/97320630005213 PMC3041001

[B42] Pan M. Santangelo T. J. Li Z. Reeve J. N. Kelman Z. (2011). *Thermococcus kodakarensis* encodes three MCM homologs but only one is essential. Nucleic Acids Res. 39, 9671–9680. 10.1093/nar/gkr624 21821658 PMC3239210

[B43] Pan M. Santangelo T. J. Cubonová L. Li Z. Metangmo H. Ladner J.. (2013). *Thermococcus kodakarensis* has two functional PCNA homologs but only one is required for viability. Extremophiles 17, 453–461. 10.1007/s00792-013-0526-8 23525944 PMC3743106

[B44] Paul S. Million-Weaver S. Chattopadhyay S. Sokurenko E. Merrikh H. (2013). Accelerated gene evolution through replication–transcription conflicts. Nature 495, 512–515. 10.1038/nature11989 23538833 PMC3807732

[B45] Pelve E. A. Martens-Habbena W. Stahl D. A. Bernander R. (2013). Mapping of active replication origins *in vivo* in thaum- and euryarchaeal replicons. Mol. Microbiol. 90, 538–550. 10.1111/mmi.12382 23991938

[B46] Quinlan A. R. Hall I. M. (2010). BEDTools: a flexible suite of utilities for comparing genomic features. Bioinformatics 26, 841–842. 10.1093/bioinformatics/btq033 20110278 PMC2832824

[B47] Robinson N. P. Bell S. D. (2005). Origins of DNA replication in the three domains of life. FEBS J. 272, 3757–3766. 10.1111/j.1742-4658.2005.04768.x 16045748

[B48] Samson R. Y. Xu Y. Gadelha C. Stone T. A. Faqiri J. N. Li D.. (2013). Specificity and function of archaeal DNA replication initiator proteins. Cell. Rep. 3, 485–496. 10.1016/j.celrep.2013.01.002 23375370 PMC3607249

[B49] Santangelo T. J. Cubonova L. James C. L. Reeve J. N. Cubonová L. James C. L.. (2007). TFB1 or TFB2 is sufficient for *Thermococcus kodakaraensis* viability and for basal transcription *in vitro*. J. Mol. Biol. 367, 344–357. 10.1016/j.jmb.2006.12.069 17275836 PMC1855253

[B50] Santangelo T. J. Cubonova L. Reeve J. N. (2008). Shuttle vector expression in *Thermococcus kodakaraensis*: contributions of cis elements to protein synthesis in a hyperthermophilic archaeon. Appl. Environ. Microbiol. 74, 3099–3104. 10.1128/AEM.00305-08 18378640 PMC2394913

[B51] Scholefield G. Veening J. W. Murray H. (2011). DnaA and ORC: more than DNA replication initiators. Trends Cell Biol. 21, 188–194. 10.1016/j.tcb.2010.10.006 21123069

[B52] Smith C. E. Llorente B. Symington L. S. (2007). Template switching during break-induced replication. Nature 447, 102–105. 10.1038/nature05723 17410126

[B53] Spaans S. K. van der Oost J. Kengen S. W. (2015). The chromosome copy number of the hyperthermophilic archaeon *Thermococcus kodakarensis* KOD1. Extremophiles 19, 741–750. 10.1007/s00792-015-0750-5 25952670 PMC4502288

[B54] Tagashira K. Fukuda W. Matsubara M. Kanai T. Atomi H. Imanaka T. (2013). Genetic studies on the virus-like regions in the genome of hyperthermophilic archaeon, *Thermococcus kodakarensis*. Extremophiles 17, 153–160. 10.1007/s00792-012-0504-6 23224520

[B55] Van der Auwera G. A. Carneiro M. O. Hartl C. Poplin R. Del Angel G. Levy-Moonshine A.. (2013). From fastQ data to high-confidence variant calls: the genome analysis toolkit best practices pipeline. Curr. Protoc. Bioinform. 43, 11.10.1–33 10.1002/0471250953.bi1110s43 25431634 PMC4243306

[B56] Wigley D. B. (2009). ORC proteins: marking the start. Curr. Opin. Struct. Biol. 19, 72–78. 10.1016/j.sbi.2008.12.010 19217277

[B57] Wu Z. Liu J. Yang H. Liu H. Xiang H. (2014). Multiple replication origins with diverse control mechanisms in *Haloarcula hispanica*. Nucleic Acids Res. 42, 2282–2294. 10.1093/nar/gkt1214 24271389 PMC3936714

[B58] Xu J. Yanagisawa Y. Tsankov A. M. Hart C. Aoki K. Kommajosyula N.. (2012). Genome-wide identification and characterization of replication origins by deep sequencing. Genome Biol. 13:R27. 10.1186/gb-2012-13-4-r27 22531001 PMC3446301

