# Supplementary material for: Genome Replication in Thermococcus kodakarensis Independent of Cdc6 and an Origin of Replication
Source: Front Microbiol. 2017 Oct 27;8:2084. doi: 10.3389/fmicb.2017.02084 (PMC5663688; doi:10.3389/fmicb.2017.02084)
Supplement: Supplementary file 1 [file Image1.PDF]

Supplementary Material

Genome replication in Thermococcus kodakarensis is not dependent on Cdc6 and does not initiate from a defined origin.

Alexandra M. Gehring[1], David P. Astling[2], Rie Matsumi[3], Brett W. Burkhart[1], Zvi Kelman[4], John N. Reeve[3], Kenneth L. Jones[2], Thomas J. Santangelo[1]*

*** Correspondence:** Thomas J. Santangelo: thomas.santangelo@colostate.edu

1 Supplementary Figures and Tables

1.1 Supplementary Table

Supplementary Table 1: Alignment Statistics

Sample	Number of Reads	Filtered Reads	Aligned Reads	Percent Aligned
TS559 exponential	67,633,173	65,914,849	65,058,090	96.2
TS559 stationary	43,521,320	42,699,034	42,102,125	96.7
Δ*cdc6* exponential	66,437,434	64,584,318	63,846,669	96.1
Δ*cdc6* stationary	67,489,844	65,733,849	65,125,236	96.5
Δ*cdc6*Δ*ori* exponential	40,886,671	40,883,270	40,475,385	99.0
Δ*cdc6*Δ*ori* stationary	49,284,832	49,278,072	48,239,873	97.9
MG1655 exponential	130,627,142	129,951,009	125,558,107	96.1
MG1655 stationary	164,618,767	163,804,543	162,897,668	99.0
JFW02 exponential	46,985,782	46,982,363	46,396,801	98.7
JFW02 stationary	42,499,179	42,493,069	33,714,141	79.3

1.2 Supplementary Figures

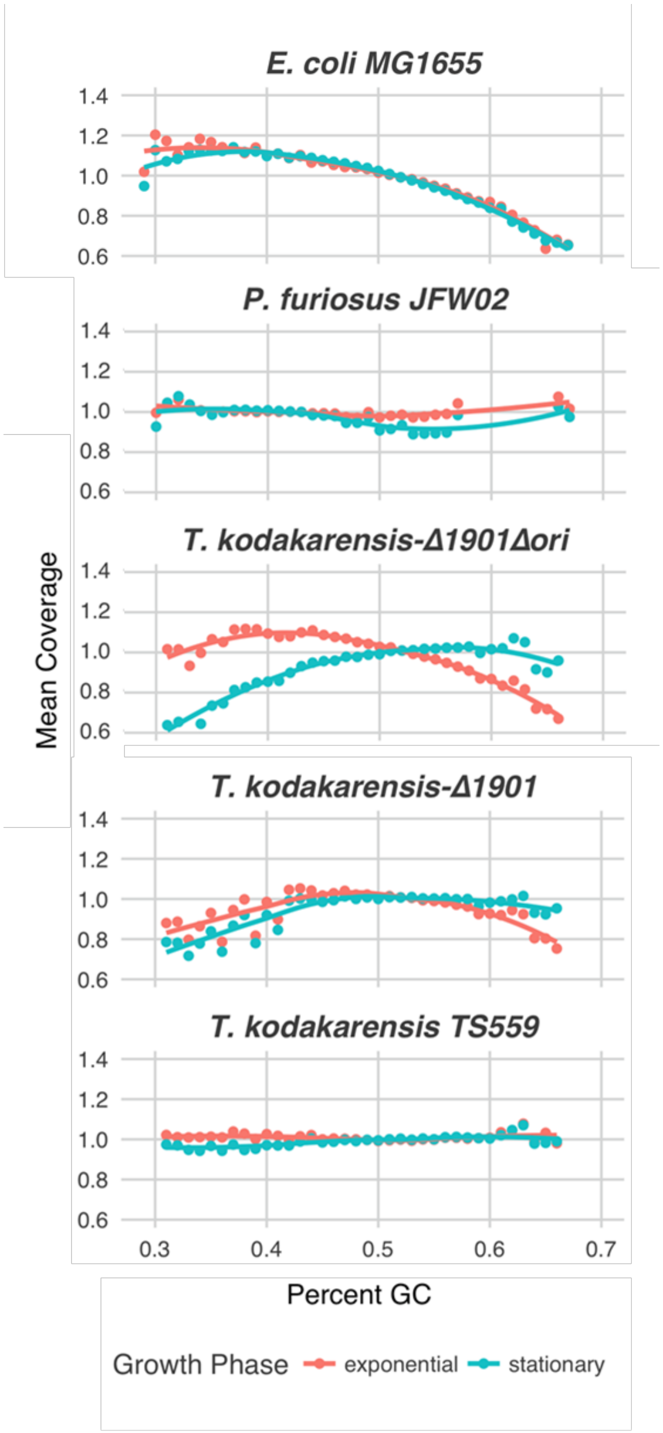

Supplementary Figure 1. GC bias of WGS data of all strains used in the copy number analysis. The normalized coverage is plotted against the %GC content for each bin for the exponential (red) and stationary (blue) cultures.

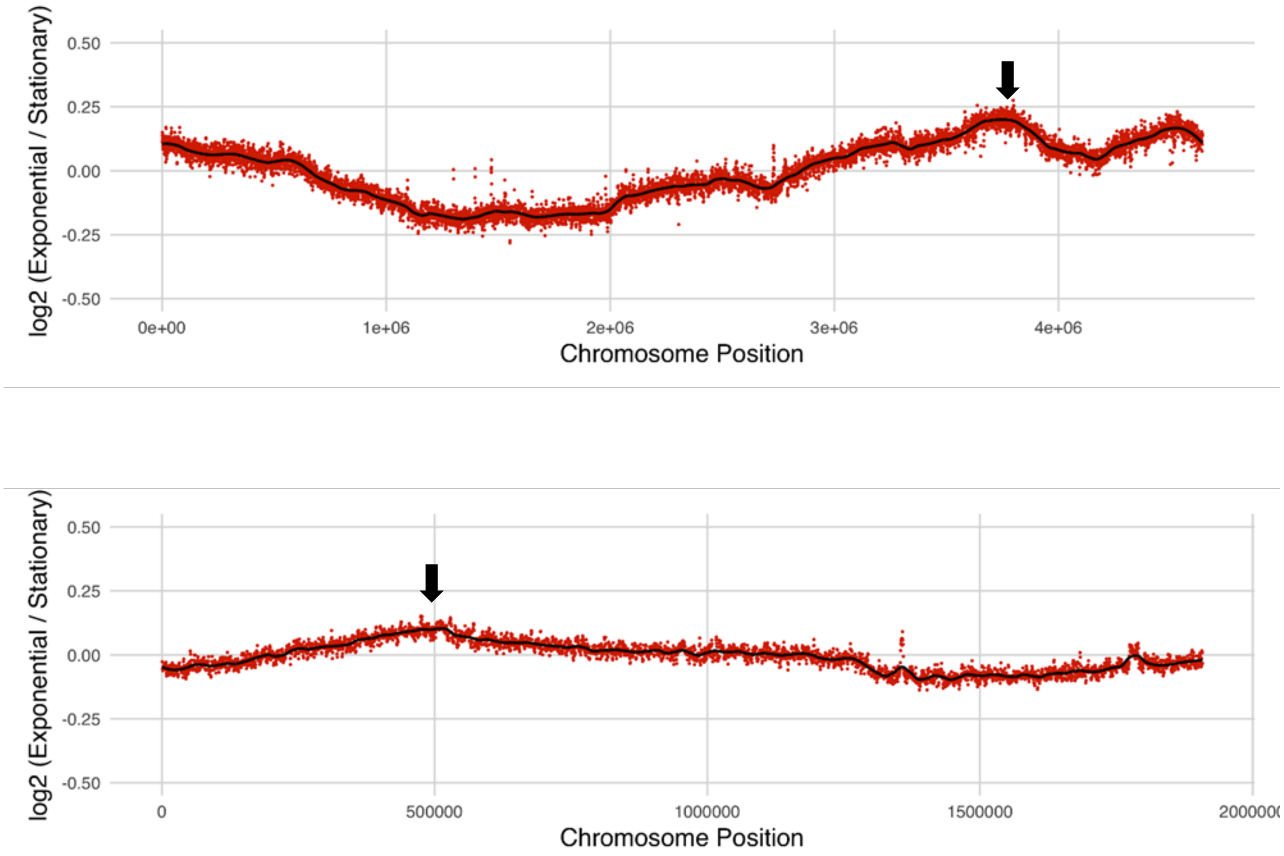

Supplementary Figure S2. Copy number analysis for the *E. coli* MG1655 (top panel) and for *P. furiosus* JFW02 (bottom panel). The log_2 frequency of each nucleotide in the WGS reads is plotted against the genome coordinates. The known, single replication origins [black arrows; (32, 33)] were identified for both species.
